# A Joint Evaluation of Impaired Cardiac Sympathetic Responses and Malnutrition-Inflammation Cachexia for Mortality Risks in Hemodialysis Patients

**DOI:** 10.3389/fmed.2020.00099

**Published:** 2020-03-27

**Authors:** Jia-Feng Chang, Chang-Chin Wu, Chih-Yu Hsieh, Yen-Yao Li, Ting-Ming Wang, Jian-Chiun Liou

**Affiliations:** ^1^Division of Nephrology, Department of Internal Medicine, Taipei Medical University-Shuang Ho Hospital, New Taipei City, Taiwan; ^2^Graduate Institute of Aerospace and Undersea Medicine, Academy of Medicine, National Defense Medical Center, Taipei, Taiwan; ^3^Division of Nephrology, Department of Internal Medicine, En Chu Kong Hospital, New Taipei City, Taiwan; ^4^Department of Nursing, Yuanpei University of Medical Technology, Hsinchu, Taiwan; ^5^Renal Care Joint Foundation, New Taipei City, Taiwan; ^6^Department of Pathology, Tri-Service General Hospital, National Defense Medical Center, Taipei, Taiwan; ^7^Department of Biomedical Engineering, Yuanpei University of Medical Technology, Hsinchu, Taiwan; ^8^Department of Orthopedics, En Chu Kong Hospital, New Taipei City, Taiwan; ^9^Department of Orthopedic Surgery, Chang Gung Memorial Hospital, Chiayi City, Taiwan; ^10^College of Medicine, Chang Gung University, Taoyuan City, Taiwan; ^11^Department of Orthopaedic Surgery, National Taiwan University Hospital, Taipei, Taiwan; ^12^School of Biomedical Engineering, Taipei Medical University, Taipei, Taiwan

**Keywords:** cardiac sympathetic response, malnutrition-inflammation score, autonomic neuropathy, protein energy wasting, mortality, hemodialysis

## Abstract

**Background:** Cardiac sympathetic response (CSR) and malnutrition-inflammation syndrome (MIS) score are validated assessment tools for patients' health condition. We aim to evaluate the joint effect of CSR and MIS on all-cause and cardiovascular (CV) mortality in patients with hemodialysis (HD).

**Methods:** Changes in normalized low frequency (ΔnLF) during HD were utilized for quantification of CSR. Unadjusted and adjusted hazard ratios (aHRs) of mortality risks were analyzed in different groups of ΔnLF and MIS score.

**Results:** In multivariate analysis, higher ΔnLF was related to all-cause, CV and sudden cardiac deaths [aHR: 0.78 (95% confidence interval (CI): 0.72–0.85), 0.78 (95% CI: 0.70–0.87), and 0.74 (95% CI: 0.63–0.87), respectively]. Higher MIS score was associated with incremental risks of all-cause, CV and sudden cardiac deaths [aHR: 1.36 (95% CI: 1.13–1.63), 1.33 (95% CI: 1.06 – 1.38), and 1.50 (95% CI: 1.07–2.11), respectively]. Patients with combined lower ΔnLF (≤6.8 nu) and higher MIS score were at the greatest risk of all-cause and CV mortality [aHR: 5.64 (95% CI: 1.14–18.09) and 5.86 (95% CI: 1.64–13.65), respectively].

**Conclusion:** Our data indicate a joint evaluation of CSR and MIS score to identify patients at high risk of death is more comprehensive and convincing. Considering the extremely high prevalence of cardiac autonomic neuropathy and malnutrition-inflammation cachexia in HD population, a non-invasive monitoring system composed of CSR analyzer and MIS score calculator should be developed in the artificial intelligence-based prediction of clinical events.

## Introduction

Interest is increasing rapidly in the application of non-invasive monitoring systems and artificial intelligence (AI)-based decision support technologies to predict clinical events in patients undergoing maintenance hemodialysis (HD), such as sudden death, emergency visit, muscle spasm, and hospitalization (1, 2). Cardiovascular (CV) diseases still top the list as the leading causes of fatal events in HD patients worldwide (3–5). Over time, the major causes of CV death shift from acute myocardial infarction and heart failure to sudden cardiac death (SCD) (6). The impaired cardiac sympathetic response (CSR) highly prevalent among HD patients is intricately involved in intradialytic hypotension and SCD (7–9). Meanwhile, diabetic autonomic neuropathy (DAN), a serious complication of diabetes mellitus (DM), plays a pivotal role in the pathogenesis of cardiac autonomic neuropathy (CAN) (10). Since end-stage renal disease (ESRD) is most often caused by DM, the heart rate variability (HRV) used for quantification of CAN serves as a valuable non-invasive monitoring tool for patient safety (11, 12).

From another point of view, the severity of malnutrition-inflammation cachexia (MIC) is in parallel with risks of all-cause and CV mortality (13). Malnutrition-inflammation syndrome (MIS) score online calculator is a validated tool in predicting composite outcomes and fatal CV events (14, 15). In light of this, electrocardiogram signals during HD and MIS score should be measured and merged into a continuous surveillance system. Nonetheless, there has been no study to evaluate the joint effect of impaired CSR and MIC on clinical outcomes in HD patients. Thus, we aimed to investigate the interaction between lower CSR and higher MIS score on death risks in this population-based study, providing preliminary data bank for future AI-based prediction models.

## Experimental Section

### Cohort

The study protocol was approved by the Institutional Review Board of the En Chu Kong Hospital (ECKIRB1041102; ECKIRB1071202). All clinical investigations were conducted according to the principles of the Declaration of Helsinki. The prospective cohort was conducted in HD patients at blood purification centers from March 2016 until February 2020. A written informed consent was obtained from the participants of this study. Patients undergoing HD treatment for at least 3 months were eligible for inclusion. All patients had to be older than 18 years of age and receive thrice-weekly HD. Patients were excluded from the study if they had terminal illness, active infections, active malignancy, or personal reasons. HD vintage was defined as the duration of time between the first day of HD treatment and the first day that the patient entered the study cohort. Each HD session was performed for 3.5–4.5 h with a blood flow rate of 200–300 mL/min and dialysate flow rate of 500 mL/min. Blood pressure was recorded in the horizontal recumbent position before dialysis session. Pre-dialysis blood samples were obtained from the existing vascular access.

### HRV Analysis for CSR

The detailed measurements and procedures for HRV analysis were previously reported (12, 16–18). All measurements for the spectral analysis were conducted in a quiet HD room. On the days of HD, all study patients received twice HRV analysis (before and after HD sessions). The protocol for HRV analysis was designed according to the standard method. After patients laid and breathed smoothly, the electrocardial signals were continuously recorded for 5 min via electrodes placed on four limbs. Cardiac autonomic signals were detected using a HRV analysis device (USPTO Application #: #20180092631) with an analog-to-digital converter. Digitized signals were analyzed online and stored in a hard disk for off-line verification. A computer algorithm was used for digital signal processing, identifying each QRS complex and excluding ventricular premature complex or noises. Stationary R-R values were re-sampled and interpolated at a rate of 7.11 Hz to produce continuity in the time domain (19). Fast Fourier transformation (FFT) was applied as a non-parametric method for frequency-domain analysis. Based on FFT, our algorithm evaluated the power spectrum density for each time segment with appropriate corrections. The power spectrum quantified by the standard frequency-domain analysis was divided into very low frequency (VLF) (0.003–0.04 Hz), low frequency (LF) (0.04–0.15 Hz), and high frequency (HF) (0.15–0.40 Hz). All HRV data were logarithmically transformed to adjust for the skewness of the distribution (16). Emergent evidences have shown patients with impaired sympathetic response during HD are at high risk of adverse clinical outcomes (8, 12). Normalized LF (nLF) has been well-described as an index of cardiac sympathetic activity: nLF (%) = LF/(total power-VLF)^*^100. Thus changes in nLF (ΔnLF) before and after HD represent CSR in HD patients (12).

### Bio-clinical Parameters and MIS Score

The following bio-demographic and laboratory parameters of each patient were recorded at baseline: age, gender, hypertension, DM, coronary artery disease (CAD), HD vintage, blood pressure, potassium, calcium, phosphorus, creatinine, pre-dialysis blood urea nitrogen, fasting glucose, alanine aminotransferase, albumin, C-reactive protein (CRP), uric acid, Kt/V (clearance of urea multiplied by dialysis duration and normalized for urea distribution volume), total cholesterol, triglyceride, high-density lipoprotein, low-density lipoprotein, iron, ferritin, total iron-binding capacity (TIBC), hemoglobin, and platelet count. We adjusted serum calcium according to the following equation: adjusted calcium = measured calcium+ [(4.0 – serum albumin in g/dL) ^*^ 0.8]. All laboratory tests were performed by standard procedures with certified methods. MIS score parameters were estimated through an online scientific calculator (http://www.touchcalc.com/calculators/mis). MIS score includes seven conventional components of the subjective global assessment (weight loss in the preceding 6 months, gastrointestinal symptoms, dietary food intake, functional capacity, comorbidities, subcutaneous fat loss and signs of muscle wasting) and three new elements (body mass index, serum albumin, and TIBC). Each MIS component has four levels of severity from 0 (normal) to 3 (very severe).

### Outcomes and Follow-Up

CV mortality in study patients was defined as death attributable to CAD, myocardial ischemia and infarction, heart failure, fatal arrhythmia, cardiac arrest because of other causes, cerebrovascular diseases, pulmonary embolism, peripheral artery diseases, and sudden otherwise unexplained death. SCD was defined by World Health Organization criteria: a sudden, natural, non-violent and unexpected death with all potentially lethal non-cardiac causes ruled out (20). Non-CV mortality was defined as all other causes of death, i.e., infection, malignancies, gastrointestinal hemorrhage, accidents, and miscellaneous. All-cause mortality included CV and non-CV death. Patients were censored if they met one of the criteria listed below during the follow-up: (1) patients were transferred to another dialysis unit; (2) patients abandoned HD treatment; (3) patients switched to peritoneal dialysis; (4) patients had recovery of kidney function and stopped HD treatment; and (5) patients received kidney transplant.

### Statistical Analysis

Statistical analysis was performed using SPSS 22.0 for windows (SPSS Inc. Chicago, USA). Continuous variables were presented as mean ± standard deviation (SD). Categorical variables were expressed as patient number (*n*) and percentage (%). The univariate Cox regression analysis was performed to investigate the independence of risk factors associated all-cause, CV mortality and SCD. The included subjects for final analysis were further stratified into higher and lower concentration groups by median values of ΔnLF and MIS score, respectively. Unadjusted and multivariable adjusted hazard ratios (aHRs) of mortality risks were calculated for different categories of ΔnLF and MIS score in the Cox regression model. The modification effect between ΔnLF and MIS score on mortality risks was determined using an interaction product term. According to methods previously described, an interaction occurs when the impact of a risk factor on outcome is changed by the value of a third variable, sometimes referred to as effect modification.

The multiplicative interaction term derived from the product of ΔnLF and MIS score acts as a third variable. We evaluated if the effect of ΔnLF on mortality risks was modified by MIS score through incorporating an interaction term in the multivariate model. The cumulative survival probability and proportional hazards were presented by graphical methods. A significant product term indicates that there is an interaction between ΔnLF and MIS score on the probability of mortality risks. A *P* < 0.05 was considered statistically significant.

## Results

The final study sample included 160 patients with maintenance HD obtaining complete medical records and follow-up. Baseline bio-clinical data of the whole study population with comparison between DM and non-DM are summarized in Table 1. The mean age was 60.7 ± 10.3 years, approximately 47% were male. Prevalence of DM, hypertension, and previous CAD was 46.3, 41.9, and 21.9%, respectively. The mean duration of follow-up was 31.9 ± 7.4 months. The overall mortality rate was 25.0% during 5097.6 person-months of follow-up, corresponding to an annual mortality rate of 9.4%. Twenty-four patients (60.0%) died from CV causes, and 16 (40.0%) were non-CV deaths. Thirteen patients (32.5%) died from SCD, and 11 (27.5%) were non-sudden CV deaths. With respect to bio-clinical parameters, there were many significant differences between DM and non-DM group. Older age and CAD history were more frequent in DM group. Furthermore, DM patients had lower levels of ΔnLF/post-dialysis nLF and higher levels of MIS score, higher pre-dialysis glucose, and phosphate. The comparisons among survivors, non-CV deaths, non-sudden CV deaths, and SCD are summarized in Table 2. The bio-clinical parameters that differed significantly included age, DM, prior CAD, ΔnLF, post-dialysis nLF, MIS score, albumin, CRP, HDL, and TIBC. It is noteworthy that subjects with SCD and non-sudden CV deaths had a higher prevalence of DM and CAD, lower levels of ΔnLF, and higher levels of MIS score.

**Table 1:** Bio-clinical data of the whole study population with comparisons between DM and non-DM groups.

**Variables**	**Overall (*n* = 160)**	**DM (*n* = 74)**	**Non-DM (*n* = **86)****
**Age (years)**	**60.7** **±** **10.3**	**63.3** **±** **10.1**	**58.5** **±** **10.0**
Male, *n* (%)	75 (46.9)	37 (50.0)	38 (44.2)
**CAD**, ***n*** **(%)**	**35 (21.9)**	**24 (32.4)**	**11 (12.8)**
Hypertension, *n* (%)	67 (41.9)	33 (44.6)	34 (39.5)
Systolic blood pressure (mmHg)	132.1 ± 21.7	131.3 ± 21.8	132.8 ± 21.8
Diastolic blood pressure (mmHg)	76.0 ± 11.7	74.7 ± 11.7	77.1 ± 11.7
Pre-dialysis nLF (%)	40.4 ± 1.9	40.4 ± 2.0	40.3 ± 1.8
**Post-dialysis nLF (%)**	**45.7** **±** **5.7**	**44.5** **±** **5.4**	**46.7** **±** **5.8**
**ΔnLF (%)**	**5.3** **±** **5.5**	**4.1** **±** **5.1**	**6.3** **±** **5.7**
**MIS score**	**6.8** **±** **1.7**	**7.3** ± **1.7**	**6.4** ± **1.7**
C-reactive protein (mg/dL)	1.1 ± 1.1	1.2 ± 1.2	1.0 ± 1.1
Albumin (g/dL)	3.8 ± 0.5	3.8 ± 0.5	3.9 ± 0.5
kt/V	1.6 ± 0.3	1.6 ± 0.3	1.6 ± 0.3
Hemodialysis vintage (months)	58.5 ± 51.9	51.4 ± 47.4	64.5 ± 55.1
**Pre-dialysis glucose (mg/dL)**	**146.3** **±** **54.3**	**187.9** **±** **43.2**	**110.5** **±** **33.4**
Alanine aminotransferase (IU/L)	15.0 ± 11.3	15.0 ± 10.1	14.0 ± 12.3
Aspartate aminotransferase (IU/L)	16.0 ± 6.4	16.4 ± 5.6	15.6 ± 7.0
Total cholesterol (mg/dL)	196.2 ± 49.5	200.1 ± 50.7	184.9 ± 44.5
Triglyceride (mg/dL)	206.6 ± 184.5	219.9 ± 197.5	167.2 ± 133.2
High-density lipoprotein (mg/dL)	52.2 ± 17.9	54.4 ± 18.6	46.1 ± 14.2
Low-density lipoprotein (mg/dL)	109.9 ± 36.6	110.1 ± 37.6	109.4 ± 33.9
Pre-dialysis BUN (mg/dL)	61.9 ± 18.1	64.3 ± 16.3	54.7 ± 21.2
Creatinine (mg/dl)	10.5 ± 1.7	10.7 ± 1.5	9.8 ± 2.0
Uric acid (mg/dL)	7.5 ± 1.3	7.5 ± 1.3	7.2 ± 1.4
Potassium (mmol/L)	4.6 ± 0.8	4.6 ± 0.8	4.6 ± 1.0
Adjusted calcium (mg/dL)	9.3 ± 0.8	9.4 ± 0.8	9.1 ± 0.8
**Phosphate (mg/dL)**	**4.5** **±** **1.5**	**4.5** **±** **1.6**	**4.2** **±** **1.2**
Calcium-phosphate product	41.3 ± 14.1	42.3 ± 14.9	38.3 ± 11.0
Iron (μg/dL)	79.7 ± 33.1	81.9 ± 34.9	73.1 ± 26.3
TIBC (μg/dL)	236.0 ± 41.9	244.6 ± 41.8	210.5 ± 30.8
Ferritin (ng/mL)	597.7 ± 304.9	572.3 ± 290.4	672.3 ± 336.9
Hemoglobin (g/dL)	10.5 ± 1.2	10.5 ± 1.1	10.7 ± 1.5
Platelet (k/μL)	195.8 ± 61.3	194.2 ± 60.4	200.9 ± 64.6

*Continuous variables were expressed as mean ± SD. Categorical variables were expressed as n (%). Boldface indicates significant p-values for trend among groups*.

*ALT, alanine aminotransferase; AST, aspartate aminotransferase; BUN, blood urea nitrogen; CAD, coronary artery diseases; CRP, C-reactive protein; DBP, diastolic blood pressure; DM, diabetes mellitus; HD, hemodialysis; HDL, high-density lipoprotein; Kt/V, clearance of urea multiplied by dialysis duration and normalized for urea distribution volume; LDL, low-density lipoprotein; MIS, malnutrition-inflammation syndrome; nLF, normalized low frequency; ΔnLF (%), changes in nLF before and after HD; SBP, systolic blood pressure; T-chol, total cholesterol; TIBC, total iron binding capacity; TG, triglyceride*.

**Table 2:** Comparison of demographic characteristics and relevant laboratory data among survivors, non-CV deaths, non-sudden CV deaths, and SCD.

**Variables**	**Survivors (*n* = 120)**	**Non-CV deaths (*n* = 16)**	**Non-sudden CV deaths (*n* = 11)**	**SCD (*n* = 13)**
**Age (years)**	**57.5** **±** **8.6**	**67.7** **±** **7.2**	**73.3** **±** **7.4**	**71.1** **±** **10.9**
Male, *n* (%)	57 (47.5)	11 (68.8)	3 (27.3)	4 (30.8)
**DM**, ***n*** **(%)**	**50 (41.7)**	**7 (42.5)**	**8 (72.7)**	**9 (69.2)**
**CAD**, ***n*** **(%)**	**13 (10.8)**	**7 (43.8)**	**8 (72.7)**	**7 (53.8)**
Hypertension, n (%)	49 (40.8)	7 (43.8)	7 (63.6)	4 (30.8)
SBP (mmHg)	132.3 ± 21.2	132.3 ± 21.8	135.4 ± 25.3	127.3 ± 24.5
DBP (mmHg)	77.2 ± 11.7	70.2 ± 8.7	75.9 ± 14.5	71.8 ± 10.8
Pre-dialysis nLF (%)	40.4 ± 1.9	39.9 ± 2.0	40.6 ± 2.1	40.6± 2.2
**Post-dialysis nLF (%)**	**48.1** **±** **3.9**	**39.0** **±** **4.1**	**39.1** **±** **2.2**	**37.5** **±** **5.5**
**ΔnLF (%)**	**7.7** **±** **3.5**	**-0.9** **±** **3.9**	**-1.6** **±** **1.7**	**-3.1** **±** **5.1**
**MIS score**	**6.3** ± **1.2**	**8.1** **±** **1.6**	**8.4** **±** **2.8**	**8.7** **±** **2.3**
**CRP (mg/dL)**	**0.7** **±** **0.9**	**2.3** **±** **0.9**	**2.3** **±** **0.7**	**2.5** **±** **1.3**
**Albumin (g/dL)**	**4.0** **±** **0.4**	**3.4** **±** **0.4**	**3.4** **±** **0.4**	**3.2** **±** **0.5**
kt/V	1.6 ± 0.3	1.6 ± 0.3	1.5 ± 0.3	1.5 ± 0.2
HD vintage (months)	58.5 ± 51.9	54.9 ± 50.5	69.3 ± 55.3	69.3± 55.3
Pre-dialysis glucose (mg/dL)	146.3 ± 54.3	143.5 ±53.9	165.4 ± 52.5	165.4 ± 52.5
ALT (IU/L)	15.4 ± 12.2	11.1 ± 6.4	11.8 ± 10.8	12.8 ± 7.3
AST (IU/L)	15.6 ± 5.7	17.9 ± 9.4	17.4 ± 6.7	15.8 ± 7.7
T-chol (mg/dL)	199.1 ± 50.4	176.7 ± 48.9	189.9 ± 41.6	199.2± 47.2
TG (mg/dL)	213.6 ± 189.8	170.3 ± 151.5	138.8 ± 98.6	244.8± 221.3
**HDL (mg/dL)**	**54.6** **±** **18.6**	**48.3** **±** **15.7**	**45.0** **±** **11.2**	**42.6** **±** **13.2**
LDL (mg/dL)	109.5 ± 37.6	96.2 ± 27.2	121.1 ± 37.3	121.5 ± 34.2
Pre-dialysis BUN (mg/dL)	64.2 ± 16.3	47.4 ± 19.6	58.9 ± 19.3	61.2 ± 23.4
Creatinine (mg/dl)	10.8 ± 1.5	9.8 ± 1.7	9.4 ± 2.3	9.9 ± 2.1
Uric acid (mg/dL)	7.5 ± 1.3	7.2 ± 1.2	7.6 ± 1.7	7.3 ± 1.5
Potassium (mmol/L)	4.7 ± 0.8	4.1 ± 0.8	4.3 ± 0.9	4.6 ± 1.0
Adjusted calcium (mg/dL)	9.3 ± 0.8	9.3 ± 0.8	9.3 ± 0.9	8.8 ± 0.7
Phosphate (mg/dL)	4.5 ± 1.6	3.8 ± 0.9	4.2 ± 1.3	4.8 ± 1.2
Calcium-phosphate product	42.1 ± 14.8	34.9 ± 8.9	39.1 ± 14.0	42.8 ± 10.8
Iron (μg/dL)	81.5 ± 35.0	73.8 ± 19.4	81.7 ± 36.4	68.4± 24.0
**TIBC (μg/dL)**	**244.1** **±** **41.6**	**213.1** **±** **28.7**	**207.6** **±** **38.7**	**214.4** **±** **36.0**
Ferritin (ng/mL)	566.5 ± 290.1	733.2 ± 364.8	650.6 ± 246.4	668.6 ± 369.8
Hemoglobin (g/dL)	10.5 ± 1.1	10.7 ± 1.2	10.7 ± 1.9	10.5 ± 1.5
Platelet (k/μL)	195.5 ± 61.0	205.4 ± 61.8	200.6 ± 79.3	183.6 ± 51.4

*Continuous variables were expressed as mean ± SD. Categorical variables were expressed as n (%). Boldface indicates significant p values for trend among groups*.

*ALT, alanine aminotransferase; AST, aspartate aminotransferase; BUN, blood urea nitrogen; CAD, coronary artery diseases; CRP, C-reactive protein; DBP, diastolic blood pressure; DM, diabetes mellitus; Kt/V, clearance of urea multiplied by dialysis duration and normalized for urea distribution volume; HD, hemodialysis; HDL, high-density lipoprotein; LDL, low-density lipoprotein; MIS, malnutrition-inflammation syndrome; nLF, normalized low frequency; ΔnLF (%), changes in nLF before and after hemodialysis; SBP, systolic blood pressure; SCD, sudden cardiac deaths; T-chol, total cholesterol; TG, triglyceride; TIBC, total iron binding capacity*.

In the univariate Cox regression analysis of prognostic factors, ΔnLF, MIS score, age, DM, CAD, albumin, CRP, HDL, ferritin, and TIBC were significantly associated with all-cause mortality (Table 3). In addition, the association between above prognostic factors and CV mortality remained significant. Table 4 unveiled that ΔnLF, MIS score, age and CAD were still strongly associated with SCD. Furthermore, we investigated possibly interesting covariates in the multivariate Cox regression model, including patient age, DM, ΔnLF, MIS score, and prior CAD. Multivariable-adjusted results demonstrated ΔnLF and MIS score were still significantly associated with all-cause and CV mortality [aHR: 0.781 (95% CI: 0.698–0.8740 and 1.333 (95% CI: 1.058–1.678), respectively] (Table 5).

**Table 3:** Univariate Cox regression analysis of prognostic factors for all-cause and CV mortality.

	**All-cause mortality**		**CV mortality**	
	**HR (95% CI)**	***p*-Value**	**HR (95% CI)**	***p*-Value**
Age	1.161 (1.113–1.211)	*p* < 0.01	1.200 (1.129–1.276)	*p* < 0.01
Male	0.977 (0.524–1.822)	*p* = 0.94	0.991 (0.618–1.984)	*p* = 0.11
DM	1.939 (1.030–3.652)	*p* < 0.05	3.135 (1.299–6.652)	*p* < 0.05
CAD	2.605 (1.676–4.297)	*p* < 0.01	3.183 (1.983–6.170)	*p* < 0.01
Hypertension	1.107 (0.594–2.065)	*p* = 0.75	1.145 (0.513–2.556)	*p* = 0.74
Four combined CSR-MIS categories	1.246 (1.156–1.343)	*p* < 0.01	1.258 (1.136–1.394)	*p* < 0.01
ΔnLF	0.727 (0.675–0.784)	*p* < 0.01	0.719 (0.651–0.793)	*p* < 0.01
MIS score	1.785 (1.546–2.060)	*p* < 0.01	1.828 (1.523–2.195)	*p* < 0.01
HD vintage	1.005 (1.000–1.010)	*p* = 0.06	1.005 (0.999–1.012)	*p* = 0.13
kt/V	0.544 (0.198–1.496)	*p* = 0.24	0.373 (0.100–1.388)	*p* = 0.14
SBP	0.998 (0.984–1.013)	*p* = 0.79	0.997 (0.978–1.016)	*p* = 0.75
DBP	0.969 (0.944–0.995)	*p* < 0.05	0.979 (0.947–1.013)	*p* = 0.22
Uric acid	0.883 (0.678-1.150)	*p* = 0.36	0.958 (0.694-1.321)	*p* = 0.79
Potassium	0.682 (0.481–1.011)	*p* = 0.06	0.876 (0.533–1.461)	*p* = 0.60
Calcium	0.758 (0.484–1.165)	*p* = 0.21	0.643 (0.361–1.144)	*p* = 0.13
Phosphate	0.895 (0.710–1.128)	*p* = 0.35	1.040 (0.905–1.343)	*p* = 0.76
CaP	0.985 (0.960–1.010)	*p* = 0.23	0.999 (0.971–1.028)	*p* = 0.96
Glucose	1.004 (0.999–1.010)	*p* = 0.11	1.006 (1.000–1.013)	*p* = 0.07
CRP	1.317 (1.085–1.849)	*p* < 0.05	1.350 (1.064–1.862)	*p* < 0.05
Albumin	0.061 (0.031–0.123)	*p* < 0.01	0.050 (0.020–0.123)	*p* < 0.01
AST	1.029 (0.987–1.074)	*p* = 0.18	1.018 (0.960–1.079)	*p* = 0.55
ALT	0.964 (0.924–1.006)	*p* = 0.09	0.973 (0.925–1.023)	*p* = 0.28
T-Chol	0.996 (0.990–1.002)	*p* = 0.23	0.999 (0.991–1.007)	*p* = 0.81
TG	0.999 (0.997–1.001)	*p* = 0.39	1.000 (0.997–1.002)	*p* = 0.69
HDL	0.969 (0.948–0.991)	*p* < 0.05	0.960 (0.931–0.990)	*p* < 0.05
LDL	1.002 (0.993–1.010)	*p* = 0.71	1.008 (0.998–1.019)	*p* = 0.12
TIBC	0.982 (0.973–0.990)	*p* < 0.05	0.981 (0.971–0.992)	*p* < 0.05
Ferritin	1.001 (1.000-1.002)	*p* < 0.05	1.001 (1.000-1.002)	*p* < 0.05
Iron	0.994 (0.984–1.005)	*p* = 0.28	0.995 (0.982–1.008)	*p* = 0.43
Hemoglobulin	1.123 (0.829–1.522)	*p* = 0.45	1.068 (0.729–1.565)	*p* = 0.73
Platelet count	1.001 (0.995–1.006)	*p* = 0.81	0.999 (0.100–1.388)	*p* = 0.62

*ALT, alanine aminotransferase; AST, aspartate aminotransferase; BUN, blood urea nitrogen; CAD, coronary artery diseases; CaP, calcium-phosphate product, which is the multiplication of serum calcium and phosphate levels; CRP, C-reactive protein; CSR, cardiac sympathetic response; DBP, diastolic blood pressure; DM, diabetes mellitus; HD, hemodialysis; HDL, high-density lipoprotein; Kt/V, clearance of urea multiplied by dialysis duration and normalized for urea distribution volume; LDL, low-density lipoprotein; MIS, malnutrition-inflammation syndrome; ΔnLF (%), changes in normalized low frequency before and after hemodialysis; SBP, systolic blood pressure; SCD, sudden cardiac deaths; T-chol, total cholesterol; TG, triglyceride; TIBC, total iron binding capacity*.

**Table 4:** Univariate Cox regression analysis of key prognostic factors for sudden cardiac death.

	**Sudden cardiac death**	
	**HR (95% CI)**	***p*-Value**
Age	1.182 (1.092–1.279)	*p* < 0.01
Diabetes mellitus	2.929 (0.901–9.519)	*p* = 0.07
Coronary artery diseases	3.489 (2.161–6.488)	*p* < 0.01
ΔnLF	0.699 (0.606–0.806)	*p* < 0.01
Malnutrition-inflammation score	1.974 (1.524–2.557)	*p* < 0.01

*ΔnLF (%), changes in normalized low frequency before and after hemodialysis*.

**Table 5:** Multivariate Cox regression analysis of key prognostic factors for all-cause and CV mortality.

	**All-cause mortality**		**CV mortality**	
	**HR (95% CI)**	***p*-Value**	**HR (95% CI)**	***p*-Value**
Age	1.047 (0.999–1.096)	*p* = 0.05	1.074 (1.002–1.150)	*p* < 0.05
DM	1.074 (0.554–2.084)	*p* = 0.83	1.738 (0.693–4.360)	*p* = 0.24
CAD	1.756 (0.863–3.575)	*p* = 0.12	2.082 (0.815–5.317)	*p* = 0.13
ΔnLF	0.778 (0.716–0.847)	*p* < 0.01	0.781 (0.698–0.874)	*p* < 0.01
MIS score	1.355 (1.126–1.631)	*p* < 0.01	1.333 (1.058–1.678)	*p* < 0.05

*CAD, coronary artery diseases; MIS, malnutrition-inflammation syndrome; ΔnLF (%), changes in normalized low frequency before and after hemodialysis*.

### Interaction Between ΔnLF and MIS Score on Mortality Risks

**Figure 1:**
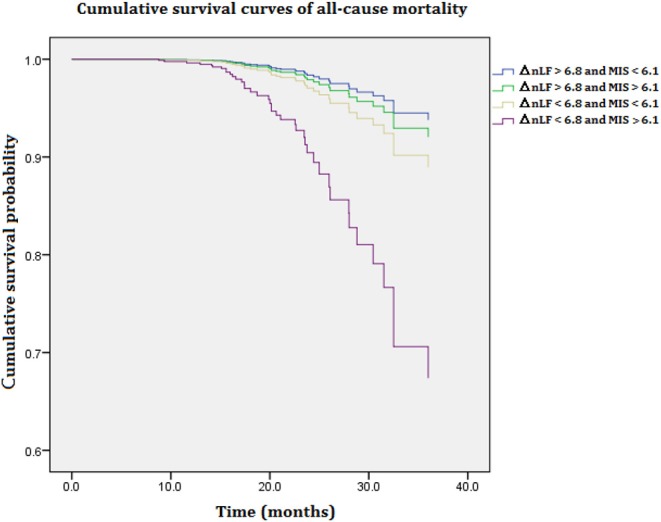
Cumulative survival curves of all-cause mortality with respect to different categories of ΔnLF and MIS score after adjusting for age, diabetes mellitus, CAD, MIS score, and ΔnLF during 5097.6 person-months of follow-up. Combined lower levels of ΔnLF (<6.8 nu) and higher levels of MIS score (>6.1 nu) was associated with a highest risk of all-cause mortality (*P* = 0.037). CAD, coronary artery diseases; MIS, malnutrition-inflammation syndrome; ΔnLF (%), changes in normalized low frequency before and after hemodialysis.

Figure 1 illustrates cumulative survival curves of all-cause mortality with respect to different categories of ΔnLF after adjusting for age, diabetes mellitus, CAD, MIS score, and ΔnLF in the Cox regression model during 5097.6 person-months of follow-up. The multivariable-adjusted result demonstrated the patient group with combined lower levels of ΔnLF (<6.8 nu) and higher levels of MIS score (>6.1 nu) was associated with a highest risk of all-cause mortality [aHR: 6.158 (95% CI: 1.120–13.843), *P* = 0.037]. Nonetheless, the *p-*value for the interaction product term was insignificant (*P* = 0.062), suggesting the association between ΔnLF and all-cause mortality was not limited to high level of MIS score.

**Figure 2:**
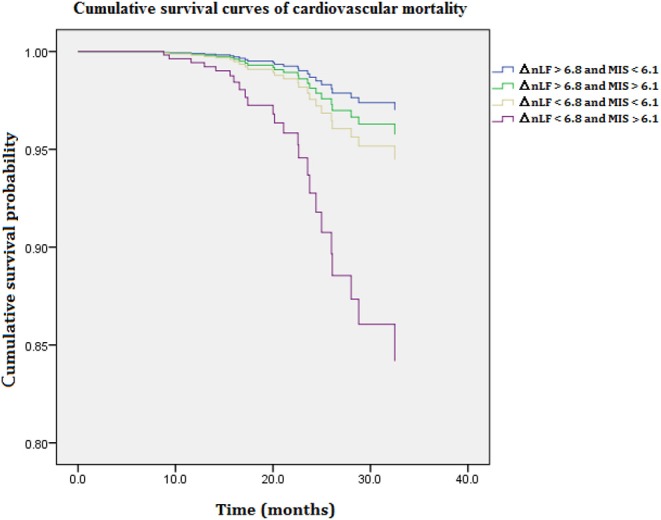
Cumulative survival curves of cardiovascular mortality with respect to different categories of ΔnLF and MIS score after adjusting for age, diabetes mellitus, CAD, MIS score, and ΔnLF during 5097.6 person-months of follow-up. Combined lower levels of ΔnLF (<6.8 nu) and higher levels of MIS score (>6.1 nu) was associated with a highest risk of cardiovascular mortality (*P* = 0.041). CAD, coronary artery diseases; MIS, malnutrition-inflammation syndrome; ΔnLF (%), changes in normalized low frequency before and after hemodialysis.

Figure 2 illustrates cumulative survival curves of CV mortality with respect to different categories of ΔnLF after adjusting for age, diabetes mellitus, CAD, MIS score, and ΔnLF in the Cox regression model during 5097.6 person-months of follow-up. The multivariable-adjusted result demonstrated the patient group with combined lower levels of ΔnLF (<6.8 nu) and higher levels of MIS score (>6.1 nu) was associated with a highest risk of CV mortality [aHR: 5.671 (95% CI: 1.060–14.708), *P* = 0.046]. Nonetheless, the *p-*value for the interaction product term was insignificant (*P* = 0.182), suggesting the association between ΔnLF and CV mortality was not limited to high levels of MIS score.

## Discussion

The services to improve patient safety are delivery of non-invasive early detection and sustained monitoring of high-risk subjects. In this prospective cohort study we present the brand new idea that the joint evaluation of ΔnLF and MIS score provides a more robust predictive value for not only all-cause but also CV mortality with the application of a non-invasive monitoring system. Our data indicate that higher ΔnLF was related to all-cause, CV and SCD [aHR: 0.78 (95% CI: 0.72–0.85), 0.78 (95% CI: 0.70–0.87), and 0.74 (95% CI: 0.63–0.87), respectively]. Higher MIS score was associated with incremental risks of all-cause, CV and sudden cardiac deaths [aHR: 1.36 (95% CI: 1.13–1.63), 1.33 (95% CI: 1.06–1.38), and 1.50 (95% CI: 1.07–2.11), respectively]. Patients with combined lower ΔnLF (≤6.8 nu) and higher MIS score were at the greatest risk of all-cause and CV mortality [aHR: 5.64 (95% CI: 1.14–18.09) and 5.86 (95% CI: 1.64–13.65), respectively]. Apparently, there is an intricate relationship between impaired CSR, MIC and fatal events. Several important findings in this work deserve further discussion.

It is of prime importance to understand the interplay between the sympathetic and parasympathetic regulatory coordination, which serves to speed up and slow down the heart rate, respectively (21). HRV is a useful non-invasive method to analyze the function of the autonomic nervous system in high-risk population. The first study of HRV in predicting mortality among 900 case of myocardial infarction patients was reported by Kleiger et al. (22). Emerging evidence supported the view that impaired CSR was a predictor of clinical events, especially for SCD (7–9, 11, 12). Thence, HRV is widely used in the medical inspection. Furthermore, HRV abnormalities have been linked to immune dysfunction and inflammation, including CV disease, diabetes, osteoporosis, arthritis, Alzheimer's disease, periodontal disease, and cancers (23, 24). To the best of our knowledge, it is the first study to investigate the interaction between impaired CSR and malnutrition-inflammation cachexia syndrome. The fact that CV disease remains the leading cause of morbidity and mortality in ESRD patients, the non-invasive measurement of ΔnLF and MIS score online scoring system should be applied and monitored in routine HD.

DAN, a serious and common complication of DM, consists of composite nerve damaging disorders (10). As the disease progresses with age, the incidence of DAN and CAN increased in DM patients (10, 25). CAN measured by HRV is strongly associated with an incremental risk of silent myocardial ischemia and death, responsible for the most critical form of DAN. Despite its relationship to death risks and multidisciplinary diseases, the significance of DAN has not been fully appreciated. Hyperglycemia is generally considered to be the major culprit of neuropathy, but the exact pathogenesis of DAN is still not clear. Elevated oxidative stress and free radicals cause vascular endothelium damages and reduce nitric oxide bioavailability (26, 27). Alternately, excess nitric oxide production may result in peroxynitrite production, endothelial damages, and neuron injuries, a process referred to as nitrosative stress (28, 29). Meanwhile, the burden of pro-inflammatory and pro-oxidant state is reminiscent of uremic milieu in chronic kidney disease (30). In light of this, ESRD patients superimposed on DM would be at the greatest risk of impaired CSR related death. Indeed, our data indicated that HD patients with DM had a lower level of CSR and higher level of MIS score (Table 1). Moreover, SCD population had the lowest level of CSR and highest level of MIS score (Table 2). In the univariate and multivariate Cox regression model, impaired CSR remained the strongest predictor of various fatal events (Tables 3–5).

In this study, the modification effect between MIS score and ΔnLF on all-cause and CV mortality was examined using an interaction product term according to previous methods of moderation analysis (31). As expected, HD patients with higher lower ΔnLF (<6.8 nu) and higher MIS score (>6.1 nu) and have the greatest risk of all-cause and CV death (Figures 1, 2). Although the association between ΔnLF and all-cause/ CV mortality was not limited to MIS score (*P* = 0.062 and 0.182 for the interaction term, respectively), our data provided a preliminary data bank for future AI-based prediction models. The majority of maintenance HD patients have CV diseases, and their CV mortality is 20 times higher than in the general population (32). The reasons why HD patients are at particular risk for CV death are complicated, e.g., ventricular hypertrophy as well as non-traditional risk factors, such as chronic volume overload, anemia, inflammation, oxidative stress, chronic kidney disease–mineral bone disorder and other aspects of the “uremic milieu.” Better understanding the effects of these numerous factors on CV death would be an important step for prevention and treatment. Accordingly, it is of prime importance to collect various aspects of clinical information from HD patients in future AI studies.

AI has influenced all aspects of human life and medical care is no exception to this growing trend. Since MIS score is a validated predictive model for various fatal events in HD patients (33, 34), we merged the online automatic MIS score calculator into the non-invasive HRV monitoring system in our study. The hope is that AI-based decision support technologies will guide multidisciplinary specialists to identify the right level of care in clinical practice, particularly in high-risk HD population. Thakur et al. successfully developed a supervised machine-learning-based prediction model to predict clinical events based on the sensor data and demographic information (1). In this manner, AI-based models help clinicians not only analyze medical data in disease prevention, diagnosis, patient monitoring and development of new protocols, but also early detect early changes to vital parameters more accurately and efficiently.

Our study has several limitations. First, the changes of electrocardiogram signals during HD and laboratory values might not reflect substantial intra-individual variability over time. Second, our sample size was relatively small and our patients were predominantly Asian patients, limiting the statistical power in the multivariate adjustment and generalization to other populations. Third, every-other-day HD preserves circadian rhythm, but a second day without HD is characterized by parasympathetic withdrawal. We did not record the electrocardiogram signals on the second day.

## Conclusions

With respect to the extremely high mortality rates in HD population, the improvement of patient safety with sustained monitoring and early detection of high-risk subjects are of prime importance. ΔnLF and MIS score predict all-cause, CV death and SCD in HD patients, yet a combination monitoring system provides more robust predictive powers. While considering the high prevalence of CAN and MIC in HD patients, ΔnLF, and MIS score could serve as a more promising dual predictor for prognostic assessment. A non-invasive monitoring system composed of HRV signal analyzer and MIS score calculator should be developed in the artificial intelligence-based prediction of clinical events.

## Data Availability Statement

The datasets analyzed in this article are not publicly available. Requests to access the datasets should be directed to Jia-Feng Chang; cjf6699@gmail.com.

## Ethics Statement

The studies involving human participants were reviewed and approved by Institutional Review Board of the En Chu Kong Hospital (ECKIRB1041102). The patients/participants provided their written informed consent to participate in this study.

## Author Contributions

J-FC and J-CL were responsible for study concept and design, interpretation of data, writing of the manuscript, study supervision, and drafting of the manuscript. C-YH assisted with methodology, investigation, biochemical, and digital analysis. T-MW, Y-YL, and C-CW were responsible for the manuscript revision.

### Conflict of Interest

The authors declare that this study received funding from Pharmofoods Medical Editing Co., Ltd. The funder had no role in study design, data collection and analysis, decision to publish, or preparation of the manuscript.
